# Metagenome Sequencing of Prokaryotic Microbiota Collected from Byron Glacier, Alaska

**DOI:** 10.1128/genomeA.00099-13

**Published:** 2013-03-21

**Authors:** Sulbha Choudhari, Sean Smith, Sarah Owens, Jack A. Gilbert, Daniel H. Shain, Roman J. Dial, Andrey Grigoriev

**Affiliations:** a Department of Biology, Center for Computational and Integrative Biology, Rutgers University, Camden, New Jersey, USA; b Argonne National Laboratory, Lemont, Illinois, USA; c Department of Ecology and Evolution, University of Chicago, Chicago, Illinois, USA; d Department of Environmental Science, Alaska Pacific University, Anchorage, Alaska, USA

## Abstract

Cold environments, such as glaciers, are large reservoirs of microbial life. The present study employed 16S rRNA gene amplicon metagenomic sequencing to survey the prokaryotic microbiota on Alaskan glacial ice, revealing a rich and diverse microbial community of some 2,500 species of bacteria and archaea.

## GENOME ANNOUNCEMENT

Of the land surface in the world, >25% is classified as a cold environment, ranging from the Arctic tundra to glacier and polar ice. Biological activity in these low-temperature habitats is generally believed to be restricted. Glacial ice has been compared to extraterrestrial cold habitats (1), and permafrost/glacial ice has been postulated to harbor the oldest microbial cells on Earth (2). Glaciers can be defined as simple, relatively closed ecosystems sustained by primary producers (e.g., photosynthetic bacteria and algae) in the snow and ice.

Because nearly all resident organisms on glaciers are single-celled and unculturable, and even the largest glacier microorganisms have been historically misidentified, the best (and perhaps the only) way to gain insight into their community structure is by a metagenomic approach. Through the Earth Microbiome Project (EMP) (http://www.earthmicrobiome.org) (3), we have performed sequencing of a snow sample from Byron Glacier in Alaska (Global Positioning System [GPS] coordinates 60.762003 N, 148.846545 W). This sample contained surface ice and snow collected in October 2011 from an avalanche cone close to sea level (depth, 2 m; elevation, 154 m); 1 liter of melted water was filtered with a 0.22-µm filter to remove larger particles. The 16S rRNA gene V4 region was amplified using the EMP standard protocols (http://www.earthmicrobiome.org/emp-standard-protocols) (4) and sequenced on an Illumina HiSeq platform, yielding 136,579 reads of 151 bp. After removing entries with a Phred score below 20 and those containing Ns, we obtained 25,018 sequences.

Using mothur (5) with the Ribosomal Database Project (RDP) (6) and SILVA (7) databases, we performed an initial phylogenetic analysis and identified 2,459 operational taxonomic units (OTUs) at a 97% identity cutoff (species level). *Proteobacteria* (~40%) was the most abundant phylum of bacteria. Other sequences were classified as follows: *Bacteroidetes* (~22%), *Firmicutes* (~12%), *Actinobacteria* (~9%), *Cyanobacteria* (~5%), *Acidobacteria* (~3%), *Verrucomicrobia* (~3%), and *Planctomycetes* (~2%). Most of the matches represented uncultured species, and yet species saturation was not reached. Despite the use of a lower-bound estimate, our initial analysis identified a much larger microbial community than was previously anticipated. For instance, an early report 45 years ago (8) listed 354 algal and cyanobacterial species, 77 fungal species, and 35 bacterial species that occur in snow. A recent metagenomic study of glacial ice in the German Alps (9) identified 72 bacterial OTUs at a 97% identity cutoff (and 108 OTUs at a 99% cutoff).

We also identified sequences of >30 archaeal species in our sample. To our knowledge, this is the first report of archaea found in a glacier metagenome in the Northern Hemisphere (9, 10). Collectively, our data reveal an unexpected richness in this temperate glacial ecosystem, which may be consequential to the warm hydrated glacial ice that extends from the Pacific Northwest to Alaska.

### Nucleotide sequence accession number.

The sequences obtained in this project have been deposited in the NCBI Short Read Archive under the accession no. SRP018522.
